# Prostate Cancer Detection and Prognosis: From Prostate Specific Antigen (PSA) to Exosomal Biomarkers

**DOI:** 10.3390/ijms17111784

**Published:** 2016-10-26

**Authors:** Xavier Filella, Laura Foj

**Affiliations:** Department of Biochemistry and Molecular Genetics (CDB), Hospital Clínic, IDIBAPS, C/Villarroel, 170, 08036 Barcelona, Catalonia, Spain; lfoj@clinic.cat

**Keywords:** prostate cancer detection, biomarker, prostate specific antigen (PSA), prostate health index, 4Kscore, PCA3 score, miRNAs, exosomal biomarkers

## Abstract

Prostate specific antigen (PSA) remains the most used biomarker in the management of early prostate cancer (PCa), in spite of the problems related to false positive results and overdiagnosis. New biomarkers have been proposed in recent years with the aim of increasing specificity and distinguishing aggressive from non-aggressive PCa. The emerging role of the prostate health index and the 4Kscore is reviewed in this article. Both are blood-based tests related to the aggressiveness of the tumor, which provide the risk of suffering PCa and avoiding negative biopsies. Furthermore, the use of urine has emerged as a non-invasive way to identify new biomarkers in recent years, including the *PCA3* and *TMPRSS2:ERG* fusion gene. Available results about the PCA3 score showed its usefulness to decide the repetition of biopsy in patients with a previous negative result, although its relationship with the aggressiveness of the tumor is controversial. More recently, aberrant microRNA expression in PCa has been reported by different authors. Preliminary results suggest the utility of circulating and urinary microRNAs in the detection and prognosis of PCa. Although several of these new biomarkers have been recommended by different guidelines, large prospective and comparative studies are necessary to establish their value in PCa detection and prognosis.

## 1. Introduction

Prostate cancer (PCa) remains a medical challenge, since it is one of the most frequently diagnosed tumors and a common cause of cancer death among men in western countries [1]. The introduction of the measurement of prostate specific antigen (PSA) in the mid-eighties of last century represented a major innovation in the management of patients with PCa. PSA, or human kallicrein 3 (hK3), is a glandular kallikrein with abundant expression in the prostate encoded by the *KLK3* gene. The sequential measurement of PSA allows the monitoring of the response to treatment and the assessment of its effectiveness. PSA is also widely used in the detection of PCa, despite its low specificity, with false positive results in patients with benign prostatic hyperplasia (BPH). Biopsy is only positive in around 25% of patients with PSA in the range between 2 and 10 μg/L. Elevated levels of PSA are particularly found in patients with enlarged prostate volume [2]. Age-specific PSA reference ranges and PSA density have been proposed to avoid this effect, increasing the specificity of PSA. Moreover, prostate volume is a key element in multivariate PCa risk calculators [3].

More recently, several studies have shown that single nucleotide polymorphisms (SNPs) in the *KLK3* gene or other related genes can influence PSA serum levels. Gudmundsson et al. [4] detected a significant association between PSA serum levels and SNPs at six loci, finding the strongest association for SNPs located near or in the *KLK3* gene. Furthermore, some of these SNPs could be associated with predisposition to PCa and PCa aggressiveness [5]. Genetic effect on PSA expression advocates a genetic correction of PSA serum levels. Unnecessary biopsies could be avoided in genetically high PSA producers, while necessary biopsies could be performed in genetically low PSA producers. Helfand et al. [6] suggested a genetically personalized interpretation of PSA serum levels to obtain more accurate results. According to these authors, genetic correction of PSA serum levels could decrease the number of potentially unnecessary biopsies by approximately 18% to 22%, improving the use of PSA as a screening tool.

A major harm of PCa screening is overdiagnosis, defined as the diagnosis of patients whose cancer never surfaces clinically during lifetime. Active surveillance is now accepted as a valuable strategy in the management of low-risk PCa, decreasing the negative effects of overdiagnosis and overtreatment. The criteria for the selection of patients for active surveillance include several variables, such as a PSA lower than 10 μg/L, a Gleason score lower than 7 and a low number of cylinders affected by the tumor [7]. However, PCa characterization based on these findings is not ideal [8]. The availability of more accurate inclusion criteria would lead to an improvement in the management of patients with early PCa.

The introduction of a new biomarker in PCa should fulfil the dual purpose of providing increased specificity and—being related to the aggressiveness of the tumor—differentiating aggressive cancers from non-aggressive cancers. Several PSA derivatives have been proposed as PCa biomarkers with this aim. The percentage of free PSA to total PSA (%fPSA) was introduced three decades ago for the detection of PCa, but this test improves clinical information only when levels reach extreme values [9].

**Figure 1:**
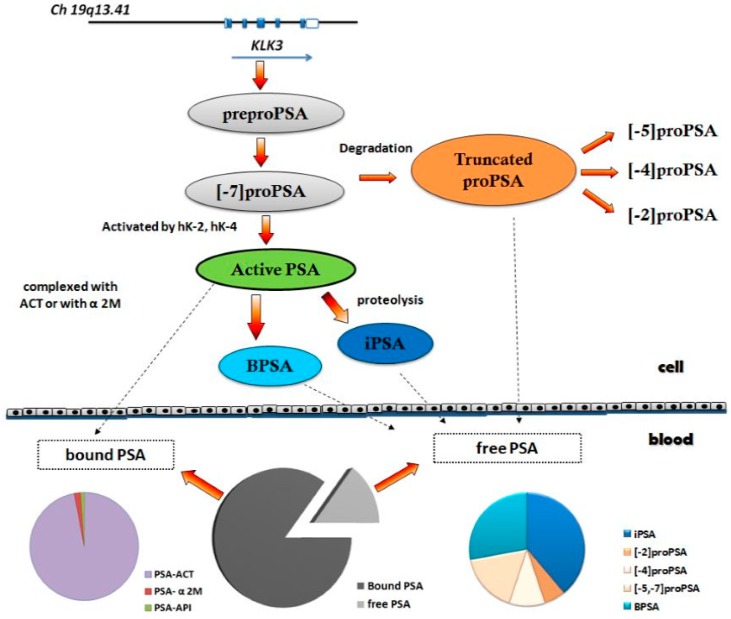
Molecular forms of PSA. The arrows with dashed line mean the forms of PSA that go from the cell to the blood. PSA: prostate specific antigen, BPSA: benign PSA, iPSA: intact PSA, PSA-ACT: Alpha 1-antichymotrypsin-PSA, PSA-API: alpha1-trypsin inhibitor PSA, PSA-A2M: alpha 2 macroglobulin, hK-2: human kallicrein 2, hk-4: human kallicrein 4.

More recently, fPSA has been found to include the isoforms BPSA, proPSA and iPSA [10] (Figure 1), with usefulness in the detection of PCa. A commercial immunoassay for the measurement of [−2]proPSA, the most stable form of proPSA, has been developed. The Prostate Health Index has been proposed as a new index for PCa detection, combining serum concentrations of [−2]proPSA, fPSA and total PSA, according to the formula ([−2]proPSA/fPSA)* √ total PSA. In addition, an index based on the measurement of total PSA, fPSA, intact PSA (iPSA) and human kallicrein 2 (hK2) in combination with clinical and demographic data, called the 4Kscore, has been proposed for the detection of high-risk PCa.

Furthermore, the development of molecular biology has allowed the study of genes associated with PCa. Numerous studies have evaluated the utility of the *PCA3* gene and more recently the *TMPRSS2:ERG* fusion gene has also been investigated. On the other hand, the study of microRNAs (miRNAs) has opened an emerging field for the research of these biomarkers in the detection and prognosis of PCa.

**Figure 2:**
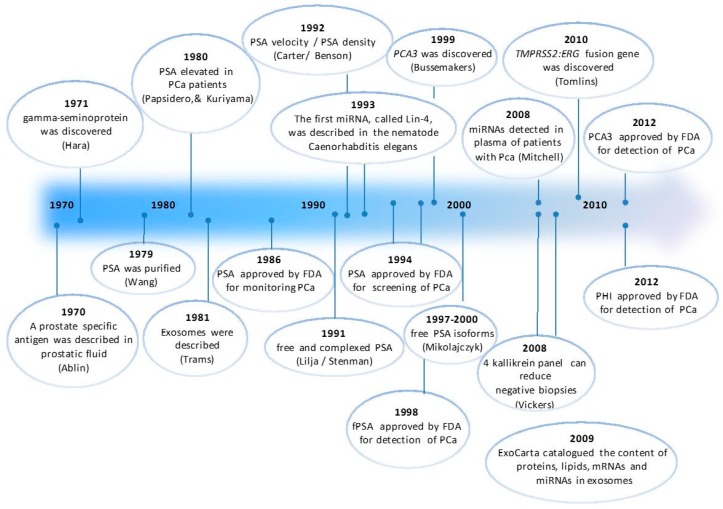
Timeline for the identification of PCa biomarkers. Most important events related to PCa markers from 1970 till the present. PSA: prostate specific antigen, miRNA: microRNA, PCa: prostate cancer, fPSA: free PSA, PHI: prostate health index.

Several sources for the study of these potential novel biomarkers for PCa are being analyzed, each one with its advantages and disadvantages. The measurement in plasma or serum has the advantage of being a consolidated media, for which commercial automated assays are available. However, the study of the *PCA3* gene in urine obtained after performing a prostate massage has opened a new way for the study of new biomarkers for PCa. Urine, due to the anatomic proximity of the prostate gland to the urethra, may allow the detection of biomarkers for PCa, particularly when the sample is enriched with prostate cells by performing a slight prostate massage. In addition, the protein content of the urine is lower than in serum and plasma, so that some interferences caused by excess of proteins can be reduced, thus facilitating the detection of new biomarkers. Finally, exosomal biomarkers obtained from blood or urine have been suggested as novel diagnostic and prognostic indicators for several cancers, e.g., PCa. Figure 2 shows a timeline for the identification of PCa biomarkers from the discovery of PSA till the current moment.

The aim of our article was to review the usefulness of blood and urine biomarkers in the detection and prognosis of PCa.

## 2. Blood-Based PCa Biomarkers

### 2.1. Prostate Health Index (PHI)

The Prostate Health Index (PHI) was approved by the Food and Drug Administration (FDA) in June 2012 for the detection of PCa in men aged 50 or older, with a PSA between 4 and 10 μg/L and a non-suspicious digital rectal examination (DRE). Also, this test is recommended by the National Comprehensive Cancer Network for patients who have never undergone a biopsy or after a negative biopsy, considering that results higher than 35 are related to a high probability of PCa [11]. Several authors agree with the usefulness of the PHI and percentage of [−2]proPSA to fPSA (%[−2]proPSA) to predict the biopsy outcome. Two multicenter studies, including 1362 and 646 patients, highlight the value of these tests in patients with PSA values between 2 and 10 μg/L. Stephan et al. [12] reported an area under the curve (AUC) of 0.72 and 0.74 for %[−2]proPSA and the PHI, respectively, while Lazzeri et al. [13] reported an AUC of 0.67 for both biomarkers.

Two meta-analyses published in 2013 and 2014 corroborate these results, documenting AUCs from 0.635 to 0.78 for %[−2]proPSA and from 0.69 to 0.781 for the PHI [14,15]. Both meta-analyses underlined that these tests outperform the results obtained with PSA and %fPSA. This point has been confirmed by a subsequent meta-analysis comparing the PHI and %fPSA in patients with total PSA between 2 and 10 μg/L [16], showing an AUCs of 0.74 and 0.63, respectively for the PHI and %fPSA.

The three mentioned meta-analyses also agree regarding the relation of the PHI and %[−2]proPSA with the aggressiveness of the tumor. This fact has been documented by Loeb et al. [17] in a study that evaluated 658 male candidates to biopsy with PSA values between 4 and 10 µg/L. The authors noted that the AUC to identify a clinically significant PCa was 0.698 for the PHI, while it was only 0.654 for %fPSA. Also, Fossati et al. [18] indicated that both the PHI and %[−2]proPSA predicted a pathologic stage T3 and a Gleason score ≥7 in the surgical specimen after evaluating a series of 489 patients treated with radical prostatectomy.

Moreover, several studies have suggested that the addition of the PHI and %[−2]proPSA to multivariate models based on the combination of PSA and various clinical and demographic variables provides better clinical performance for the prediction of PCa. Stephan et al. [19] reported that the AUC increased from 0.69 to 0.75 when [−2]proPSA or the PHI were added to a multivariable model based on patient age, prostate volume, DRE, PSA and %fPSA. Similarly, Guazzoni et al. [20] showed an improvement in the AUC by including %[−2]proPSA (0.82) or the PHI (0.83) in a model based on the patient age, prostate volume, PSA and %fPSA (0.72). Finally, Filella et al. [21] showed that the AUC increased from 0.762 to 0.802 (using a logistic regression analysis) or 0.815 (using an artificial neural network) when the PHI and %[−2]proPSA were included in a multivariate model based on patient age, prostate volume, PSA and %fPSA. Moreover, this study showed a relationship between prostate volume and the PHI values, highlighting that prostate volume is a key factor in the interpretation of PHI results. According to these authors, the performance of the PHI changed in relation to prostate volume, finding AUCs of 0.818, 0.716 and 0.654 for patients with small, medium and large prostate volume, respectively.

Lughezzani et al. [22] developed a PHI based nomogram for predicting PCa evaluating 729 patients undergoing prostate biopsy. The PHI increased significantly the accuracy obtained using a multivariable logistic regression model based on patient age, prostate volume, DRE and biopsy history from 0.73 to 0.80. External validation of this model was provided by a multicenter European study based on 833 patients, showing an accuracy of 0.752 [23]. More recently, Roobol et al. [24] showed analogous results validating this nomogram on 1185 men from four European sites, obtaining an accuracy of 0.75 for all PCa and 0.69 for clinically relevant PCa (clinical stage > T2b and/or a biopsy Gleason score ≥ 7). Similar results were found in this study by adding the PHI to the European Randomized Study of Prostate Cancer (ERSPC) risk calculator, obtaining an accuracy of 0.72 for all PCa and 0.68 for clinically relevant PCa. Despite these positive results, the authors concluded that only limited reductions in the rate of unnecessary biopsies are possible when models are updated adding the PHI. More optimistic are the conclusions of a recent cost-effectiveness study of PCa detection using the PHI [25]. The authors used a micro simulation model based on the results of the ERSPC trial to compare the effects of screening using the PHI and those using only PSA. The model predicted a reduction of 23% in negative biopsies for men with PSA between 3 and 10 μg/L, concluding that PHI testing is 11% more cost-effective than screening only based on PSA.

### 2.2. Kallikrein Panel

The 4Kscore is a risk calculation for the detection of PCa on the biopsy based on the measurement of a 4-kallikrein panel combined with the patient age, DRE and biopsy history. The 4-kallikrein panel includes the measurement of total PSA, fPSA, iPSA and hK2, a kallikrein with high homology with PSA. The test provides information about the probability of having a high-risk PCa. Therefore, the aim is no longer to indicate a biopsy to detect PCa, but it is intended only to detect aggressive tumors with a Gleason score of 7 or higher. Although the 4Kscore does not have the approval of the FDA, in June 2015 the National Comprehensive Cancer Network recommended the use of this test for the detection of high-risk PCa for patients who have never undergone biopsy or after a negative biopsy [11].

The test was developed after studies performed by the group led by Lilja and Vickers, from Memorial Sloan-Kettering Cancer Center, who conducted an extensive research in collaboration with several European centers. The AUCs for the 4-kallikrein panel reported in all these studies for the detection of high-risk PCa were higher than those for a PSA based model [26,27,28,29,30,31,32]. The AUCs obtained for the 4-kallikrein panel ranged between 0.793 and 0.870, while the AUCs for the PSA based model ranged from 0.658 to 0.816. Similar differences were observed when the corresponding clinical models to predict high-risk PCa were compared. The AUCs for the model combining PSA, patient age and DRE ranged from 0.709 to 0.868, while the AUCs ranged from 0.798 to 0.903 when fPSA, iPSA and hK2 were also included.

Prostate volume is a crucial factor influencing PSA serum levels and it is usually included in PCa risk calculators. However, according to results published by Carlsson et al. [33], prostate volume is not affecting the performance of a 4-kallicrein panel in PCa detection. This group studied these biomarkers in two cohorts of 2914 and 740 patients with PSA ≥3 μg/L. In the first cohort, the AUC increased from 0.856 to 0.860 when the prostate volume was added to a model based on a 4-kallikrein panel, patient age and DRE, while in the second cohort the AUC was identical including or not prostate volume (0.802).

All results published about the 4Kscore before 2014 were obtained in retrospective European cohorts. In 2015, a prospective multicenter study developed in United States was published [34]. This evaluation, which included 1012 patients scheduled for prostate biopsy, confirmed the results shown in previous studies. The AUC obtained for the 4Kscore test in the detection of high-risk PCa was 0.82. The study documented that 58% of biopsies could be saved using a cut-off value of 15%, although 4.7% of high-risk PCa would not be detected. Using a cut-off value of 6%, the reduction in the number of biopsies was of 30%, but in contrast, only 1.3% of high-risk tumors were not detected.

On the other hand, a study based on a cohort collected in Sweden since 1986, in which samples of included subjects were collected at the age of 40, 50 and 60, showed the predictive value of the 4Kscore [35]. The result of this test assessed at 50 and 60 years old allowed the classification of the patients into two groups according to the probability of developing distant metastasis 20 years later. Patients with PSA ≥3 μg/L at 60 years old were classified using a cut-off for the 4Kscore of 7.5 in two groups with a significantly different risk of developing distant metastasis. Similarly, the 4Kscore was also predictive using a cut-off of 5 considering patients with PSA ≥2 μg/L at 50 years old.

## 3. Urine-Based PCa Biomarkers

### 3.1. PCA3

**Figure 3:**
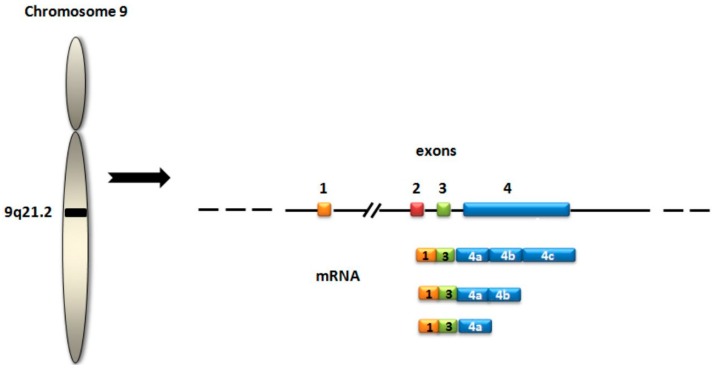
*PCA3* gene structure. The most frequent transcript contains exons 1, 3, 4a, and 4b.

*PCA3* (prostate cancer gene 3, previously referred as *DD3*) is a gene that transcribes a long non-coding mRNA that is overexpressed in PCa tissue (Figure 3). The PCA3 test is the score calculated measuring the concentration of *PCA3* mRNA in relation to PSA mRNA, which is used to normalize *PCA3* signals. Measurements are performed in the urine obtained after performing a prostate massage to enrich prostate cell content. This test, based on quantitative real time polymerase chain reaction (qRT-PCR) technology, obtained the European Conformity in November 2006 and was approved by the FDA in 2012 with the aim of deciding the repetition of a prostate biopsy in men more than 50 years old who have one or more previous negative biopsies and results of PCA3 score higher than 25. The 2015 clinical guide of the National Comprehensive Cancer Network for the early detection of PCa [11] considered PCA3 score as a useful test to decide the repetition of a biopsy in patients with a previous negative biopsy, suggesting the use of 35 as the discriminating value.

The available results suggest that the PCA3 score can be useful in detecting PCa, especially in patients with a previous negative biopsy [36]. However, the definition of the best discriminating value is controversial. A multicenter study led by Haese [37] indicated that a score of 35 provides the optimal balance between sensitivity (47%) and specificity (72%). Similar results have been recently reported in a meta-analysis published by Hu et al. [38] including 16 studies that used 35 as the cut-off to decide when to perform a biopsy. These authors documented an overall sensitivity of 57% and an overall specificity of 71%, concluding that, although high quality further studies are missing, the PCA3 score is a useful test in the detection of PCa. However, a large group of tumors were not diagnosed when a biopsy was indicated in patients with a PCA3 score higher than 35. In the aforementioned study of Haese et al. [37], 67% of biopsies would have been saved using the discriminating value of 35, but 21% of high-grade PCa would not have been diagnosed. In the same study, using a discriminating value of 20, the saved biopsies were only from 44%, but 9% of high-grade PCa were not detected.

These data have been confirmed by Crawford et al. [39] in a multicenter study involving 1913 patients, 802 of which with PCa. These authors point out that the traditional score of 35 allowed a reduction of 77% in biopsies, but the number of false negatives was large, because PCa was detected in 413 of 1275 patients (32%) with a PCA3 score lower than 35, of which 195 had a Gleason score of 7 or higher. However, by lowering the discriminating value to 10, the number of undiagnosed tumors was reduced to 108, of which 52 had a Gleason score lower than 7. This study also points out that PCa was only detected in 86 of 114 patients with a PCA3 score ≥100. It is somewhat paradoxical because initial studies presented *PCA3* as an overexpressed gene in PCa tissue between 10- and 100-fold relative to non-neoplastic tissue [40]. In this way, Schröder et al. [41] showed a low positive predictive value (38.9%) for a PCA3 score ≥100, even when significant efforts to detect a PCa were performed.

Finally, the relationship between PCA3 score and the aggressiveness of the tumor is also controversial. Merola et al. [42], in a group of 114 patients with PCa, observed a significant association between the PCA3 score and Gleason score (*p* = 0.02). Moreover, Chevli et al. [43] in a study based on 3073 patients observed that the PCA3 score is related to the Gleason score, with mean values of 47.5 and 58.5 for patients with a Gleason score of 6 and 7 or higher, respectively. However, this study also shows that the AUC to predict high-grade PCa is lower than that obtained with PSA (0.679 vs. 0.682, respectively). Other authors do not find any correlation between the PCA3 score and Gleason score, without explanations for this divergence [44,45].

The accumulated data on the PCA3 score generate an important expectation. Nevertheless, the available evidence about its usefulness is not enough, as noted by a comparative analysis published by Bradley et al. [46] based on 34 observational studies. The majority of studies suggest that the diagnostic accuracy of the PCA3 score is clearly superior to that of PSA. In this regard, however, Roobol et al. [47] highlight the influence of the bias caused by the use of PSA in the selection of the population included in *PCA3* studies. In their report, the authors try to minimize this bias by not only performing the biopsy when PSA was greater than 3 μg/L, but also when the PCA3 score was lower than 10. The obtained data showed less difference in the AUCs than in other studies (0.581 and 0.635 for PSA and PCA3 score, respectively).

### 3.2. TMPRSS2:ERG Fusion Gene

**Figure 4:**
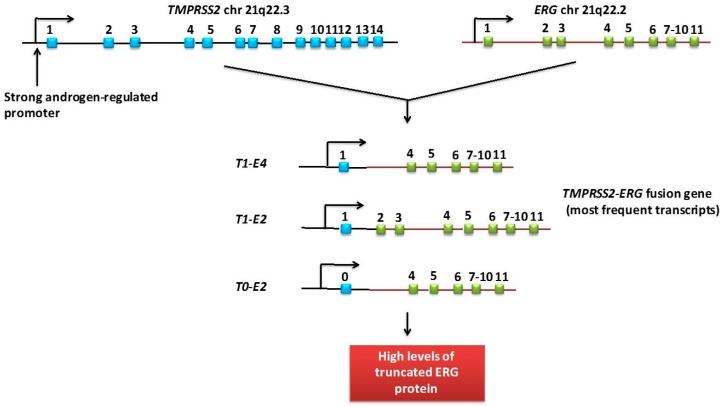
The *TMPRSS2:ERG* fusion gene and its most frequent transcripts. The most frequent transcripts of the *TMPRSS2:ERG* fusion gene consist in the fusion of exon 1 of *TMPRSS2* to either exon 2 or 4 of *ERG* (T1-E4 or T1-E2). These transcripts encode for N-terminal truncated ERG proteins. Exon 1 from *TPRSS2* is non-codifying. Transcription of the gene *TMPRSS2* can start not only from its first exon, exon 1, but also from an alternative first exon (exon 0).

The gene fusion involving *ERG* (v-ets erythroblastosis virus E26 oncogene homologs) and the androgen regulated gene *TMPRSS2* (transmembrane serine protease isoform 2) (Figure 4) was reported in 2005 by Tomlins et al. [48]. Aberrant fusion of the *TMPRSS2* gene with the *ERG* gene is observed in 15%–59% of PCa [49], but there are not unanimous results regarding its association with disease prognosis. Differences could be explained in relation to the exon involved in the fusion [50] or due to the presence of multiple copies of the fusion gene, showing a reduced prostate cancer-specific survival in those patients whose tumor had multiple copies of the fusion gene [51].

The *TMPRSS2:ERG* gene rearrangements are studied in urine samples obtained after a prostate massage using qRT-PCR. A ratio with PSA mRNA is used to normalize *TMPRSS2:ERG* signals. The combination of the TMPRSS2:ERG score with the PCA3 score has been proposed as a way to improve the prediction of the presence of PCa on the biopsy. A multicenter study published by Leyten et al. [52] prospectively evaluated the diagnostic utility of PCA3 and TMPRSS2:ERG scores in 443 patients who underwent a biopsy and found that both scores significantly increased the predictive value obtained with the risk calculator ERSPC, which includes PSA and several clinical variables. The AUC increased from 0.799 to 0.833 for the risk calculator ERSPC when PCA3 score was added, and to 0.842 when PCA3 and TMPRSS2:ERG scores were added. In addition, TMPRSS2-ERG, but not PCA3, was associated with the Gleason score and the tumor clinical stage.

More recently, Tomlins et al. [53] also proposed the combination of PCA3 and TMPRSS2:ERG scores with PSA serum levels as a useful tool for detecting PCa. The study, prospectively conducted in three centers and based on 1244 patients who underwent biopsy, showed the value of PCA3 and TMPRSS2:ERG scores when they were added to the PCPT (Prostate Cancer Prevention Trial) risk calculator, also based on PSA and several clinical variables. The AUC increased from 0.639 for the PCPT risk calculator to 0.739 and to 0.762 by adding the TMPRSS2:ERG score alone or the TMPRSS2:ERG score plus the PCA3 score, respectively. Both biomarkers were also evaluated to predict high-risk PCa, being the respective AUCs of 0.707 (for the PCPT risk calculator), 0.752 (plus *TMPRSS2:ERG*) and 0.779 (plus *TMPRSS2:ERG* and PCA3). The model that brings together the PCPT risk calculator, PCA3 and TMPRSS2:ERG scores, called My Prostate score (MiPS), would allow the avoidance of 36% of biopsies using a discriminating value of 15%, although it would cease to diagnose 1.6% of high-risk tumors.

The value of MiPS has been questioned by Stephan et al. [54] comparing PCA3, *TMPRSS2:ERG*, PSA and PHI in a series of 246 patients, including 110 patients with PCa. The authors obtained an AUC for MiPS of 0.748. The highest accuracy was found for a model including PCA3 and the PHI, with AUCs of 0.757 and 0.752, when an artificial neural network and the logistic regression analysis were used, respectively. The authors argued that the clinical potential of the TMPRSS2:ERG score is limited because of its low prevalence in PCa patients.

## 4. miRNAs

miRNAs are small (17–22 nucleotides) non-coding RNAs, which negatively regulate the gene expression at the posttranscriptional level by base-pairing to the complementary sites in their target mRNAs, resulting in a repression or degradation of the target. The number of identified miRNAs has progressively increased since 1993, when the first miRNA was described in the nematode *Caenorhabditis elegans*. Currently, the number of miRNAs described according to the database mirbase.org is 28,645 miRNAs (miRBase, release 21 June 2014) [55], 2588 of which are found in humans, targeting the vast majority of mRNAs.

**Figure 5:**
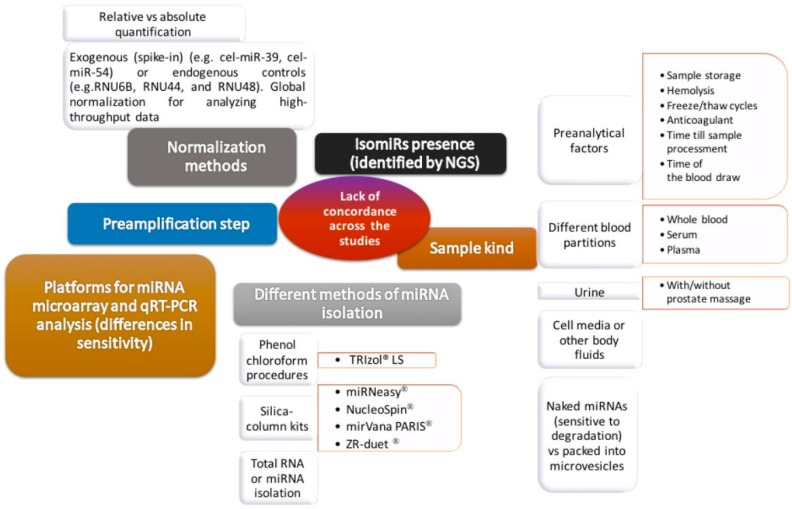
Sources of lack of concordance across the different studies. miRNA: microRNA, NGS: next generation sequencing.

Several studies have shown their participation in the development and progression of cancer. Calin et al. [56] showed in 2002 that some miRNAs were suppressed or downregulated in chronic lymphocytic leukemia. The number of studies that evaluate their usefulness as biomarkers has been increasing since 2008, when Lawrie et al. [57] proposed a profile of circulating miRNAs as a diagnostic tool in patients with B-cell lymphoma. Many research findings prove the usefulness of circulating miRNAs in the detection and prognosis of various tumors and in the prediction of the response to treatment, revealing robust signatures of miRNAs that distinguish healthy controls from patients with cancer. However, these results should be contextualized because of differences in the methodology used in the available studies. Witwer [58] underlines that limited overlap has been observed between the findings of similar studies in the same disease, probably due to methodological reasons. Indeed, although miRNAs are stable enough in plasma and serum, optimization and standardization of the methodology used for the miRNAs measurement is required to obtain high quality results. So far, there are still differences due to the type of sample (with differences between serum and plasma, and also related to plasma, among the different anticoagulants); the specific reagent used in the isolation of miRNA; the performance of a preamplification to obtain more Cdna; and the platform used for the qRT-PCR (AbiPrism, LightCycle, etc.), the use of endogenous (none of which behaves as ideal control) or exogenous controls (e.g., cel-miR-39) to normalize the results, and the calculation method used, of which probably ΔΔ*C*_t_ is the most frequent (Figure 5).

The development of digital PCR could improve the performance of qRT-PCR and would remove the dependence for a reference miRNA for normalization. Besides, the introduction of digital count technologies, such as next-generation sequencing (NGS) and the NanoString nCounter System has supplied new tools for miRNA profiling [59]. As opposed to microarrays and qRT-PCR, NGS enables the discovery of new miRNAs in addition to the confirmation of known miRNAs. It beats the limitations of microarrays that have background signal and cross-hybridization problems. Another recent novelty for miRNA profiling is the NanoString nCounter system, a hybridization-based technology, which consists on direct digital detection of RNA molecules of interest using target-specific, colour-coded probe pairs without the requirement of reverse transcription or cDNA amplification.

The expression of aberrant miRNAs has been demonstrated in PCa, playing a critical role in tumor initiation, development and progression [60]. Mitchell et al. [61] were the first to show that miRNAs are present in the plasma of patients with PCa in a remarkably stable manner, noting that miR-141 is significantly elevated in patients with advanced PCa compared to healthy controls. Subsequently, several studies based on microarrays have identified several miRNAs signatures with utility in the diagnosis and prognosis of PCa. However, the differences among these panels are substantial and only miR-141, miR-375 and miR-21 repeatedly appear in various studies [62]. Prominently, Mihelich et al. [63] retrospectively studied the serum levels of 21 miRNAs in 100 PCa patients in stages T1–T2. The authors found a signature composed by 14 miRNAs (let-7a, miR-24, miR-26b, miR-30c, miR-93, miR-100, miR-103, miR-106a, miR-107, miR-130b, miR-146a, miR-223, miR-451, and miR-874) to distinguish accurately (negative predictive value of 0.939) high-grade PCa from low-grade PCa. Seven of these miRNAs (miR-451, miR-106a, miR-223, miR-107, miR-130b, let-7a and miR-26b) were also significantly lower in PCa patients with biochemical recurrence after radical prostatectomy compared with those without biochemical recurrence. Similarly, Chen et al. [64] identified, in plasma, a panel of five miRNAs (miR-622, miR-1285, let-7e, let-7c, and miR-30c) to discriminate CaP from BPH and healthy controls with high sensitivity and specificity. On the other hand, Moltzahn et al. [65] found AUCs from 0.812 to 0.928 for miR-106a, miR-1274, miR-93, miR-223, miR-874, miR-1207 and miR-24, showing a trend to correlate with the CAPRA score, a PCa risk assessment based on patient age, PSA serum levels, clinical tumor stage, the Gleason score and the percentage of positive biopsy cores. The authors reported the up-regulation of miR-30c, miR-93, miR-106a, miR-223 and miR-451 in patients with high-risk PCa compared with low-risk PCa. It should be noted that opposite results have been indicated by Mihelich et al. [63], showing that these miRNAs were highly expressed in BPH and low-risk PCa compared with high-risk PCa. These differences could be attributed to deficiencies in the standardization of the collection and measurement of miRNAs.

Although the clinical usefulness of miRNAs in urine has been investigated by several authors, no comprehensive evaluation has been reported till the present moment. Studies have been done in several urine fractions, including whole urine, urinary pellet and the cell-free urinary fraction. The enrichment with prostatic cells after a prostate massage has been also assayed. Preliminary results suggest that the measurement of urinary miRNAs may be valuable as biomarkers in the management of patients with early PCa, although conclusions of the available studies are based on a short series of patients [62]. Recently, Salido-Guadarrama et al. [66] identified a miR-100/200b signature in the urine obtained after prostate massage comparing 73 patients with high-risk PCa and 70 patients with BPH. The AUC for this signature (0.738) was higher than the obtained AUCs for total PSA (0.681) and %fPSA (0.710). Adding the miR-100/200b signature to a multivariate model based on age, DRE, total PSA and %fPSA the AUC increased from 0.816 to 0.876.

Contributions regarding the clinical usefulness of miRNAs in blood and urine are summarized in Table 1.

## 5. Exosomal Biomarkers

Exosomes are small (30–150 nm) double lipid membrane vesicles of endocytic origin secreted by most cell types, that contain proteins, lipids and nucleic acids. Information about the molecules identified in exosomes from multiple organisms is provided in the ExoCarta database (http://exocarta.org/). Currently, ExoCarta contains information on 41,860 protein entries, 4946 mRNA entries and 2838 miRNA entries that have been identified from 286 exosomal studies. Exosomes were initially described by Trams et al. [67] as exfoliated membrane vesicles, obtained from neoplasic cell cultures. Their presence has later been confirmed in serum and plasma as well as in other biological fluids, including urine, saliva, ascites, breast milk and amniotic fluid. The study of new biomarkers in exosomes is a promising field because they are remarkably stable in body fluids and their content is protected from enzymatic degradation by the exosomal lipid bilayer [68,69].

Cancer cells have been shown to release high levels of exosomes, which may influence tumor initiation, growth and progression as well as drug resistance. Actually, a significantly higher amount of exosomes has been found in serum from ovarian cancer patients in comparison with healthy subjects or patients with benign ovarian diseases. Also, the amount of exosomes has been correlated with the clinical stage in ovarian cancer, showing high levels in patients at an advanced stage [70]. Duijvesz et al. [71] showed that CD9 and CD63 exosomal biomarkers in urine collected after DRE were significantly higher in men with PCa. Analogous results have been found in other tumors, including lung cancer, colorectal cancer and chronic lymphocytic leukemia [72,73,74].

The emerging involvement of exosomes in intercellular communication focuses on their role in carcinogenesis. Tumor derived exosomes exchange information with other cancer cells and also with other cell types, including stroma and extracellular matrix cells, establishing favorable conditions for tumor growth and invasion. Exosomes can stimulate target cells through different ways, including the interaction with specific membrane receptors, endocitosis and the horizontal transfer of proteins and RNA species, such as mRNA, large non-coding RNA molecules and miRNAs [75]. Available evidence showed that exosomes derived from cancer cells actively contribute to the progression of disease [76]. Franzen et al. [77] reported that epithelial-to-mesenchymal transition in urothelial cells is induced by muscle invasive bladder cancer exosomes. Similar data have been shown in PCa. Abd Elmageed et al. [78] demonstrated that exosomes from PCa cells can induce the neoplastic transformation of adipose derived stem cells through the horizontal transfer of several oncogenic factors, including the oncomiRs miR-125b, miR-130b and miR-155. Besides, according to Hosseini-Beheshti et al. [79], exosomes derived from PCa cells could contribute to cancer progression, reducing apoptosis, increasing cancer cell proliferation, and inducing cell migration in LNCaP and RWPE-1 cells.

**Figure 6:**
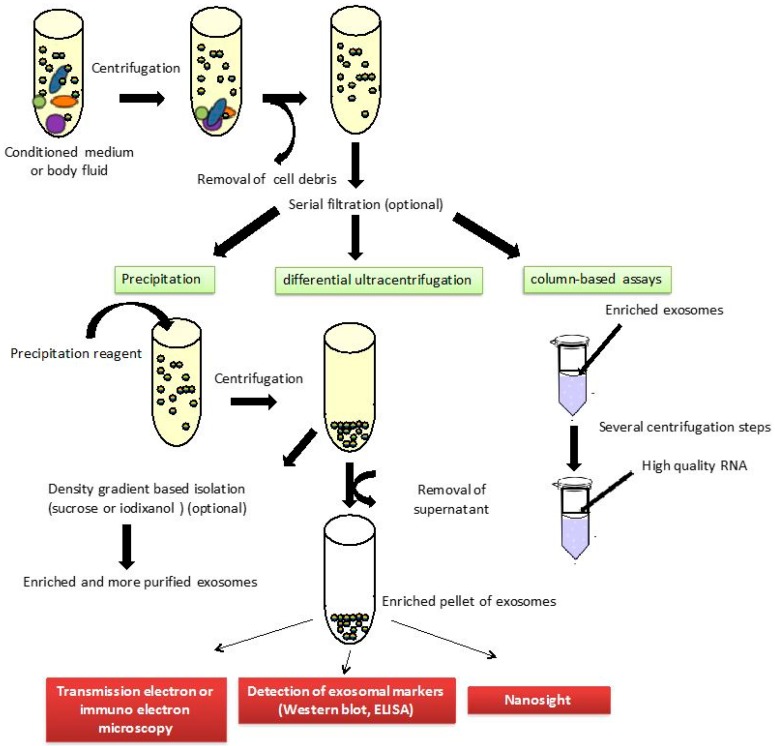
Exosome isolation procedures. The different size particles contained in body fluids or conditioned media are drawn as different color circles.

In spite of the growing interest in the role of exosomes in several diseases and their use for functional studies and biomarker discovery, no standard method is available to acquire highly pure and well-characterized exosomes. The most common method for exosomes isolation is differential ultracentrifugation, which consists of multiple centrifugation steps with increasing centrifugal strength to sequentially pellet cells (300 g), microvesicles (10,000 g) and exosomes (100,000 g) [80]. Many variations to these speeds are implemented in practice. Serial filtration through 0.45 and 0.2 mm filters is used optionally before exosome pelleting. Furthermore, density gradient based isolation, using sucrose or iodixanol (OptiPrep™, Sigma-Aldrich, St. Louis, MO, USA), can be used to obtain more pure exosome preparations. Commercial easy-to-use reagents have recently been developed, including precipitation solutions, such as ExoQuick™ or Total Exosome Isolation™ kits, and column-based assays, such as exoRNeasy Serum/Plasma. Transmission electron microscopy or immuno electron microscopy can be performed to check the presence of exosomes. Furthermore, Western blot and ELISA assays can be used to detect several exosomal markers (such as CD9, CD63 and CD81) in order to identify the isolated vesicles as exosomes (Figure 6).

Few studies have evaluated exosomal biomarkers in PCa detection and prognosis. Proteome of urinary exosomes has been analyzed using mass spectrometry to identify proteins by Øverbye et al. [81] in 15 healthy subjects and 16 PCa patients. This study showed that 246 proteins were significantly altered in urinary exosomes of PCa patients. The authors found that 17 proteins presented sensitivities above 60% at 100% specificity. The highest sensitivities were observed for transmembrane protein 256 (TM256) (94%), LAMTOR1 (81%) and ADIRF (81%). Furthermore, the authors reported an AUC of 0.87 for TM256, which increased to 0.94 by combining with LAMTOR1.

Comparison of *PCA3* and *TMPRSS2:ERG* between urinary sediment and exosomes has been reported by Dijkstra et al. [82], concluding that exosomes seem to be a more robust source of biomarkers, although a significant proportion of samples are not assessable because they do not reach the analytical detection limit. Exosomal *PCA3* and *TMPRSS2:ERG* levels were significantly higher when urine was collected after a prostate massage. More recently, published results by this group based on 29 men undergoing prostate biopsies showed that *PCA3* and *ERG* perform better in whole urine than in urinary sediment or exosomes [83]. No significant differences were found for both biomarkers in exosomes comparing PCa and non-PCa patients.

Opposite results have been reported by Donovan et al. [84] evaluating the combination of exosomal *PCA3* and *ERG* mRNA in the detection of high-grade PCa. The authors studied both biomarkers in first-catch urine samples obtained without prostate massage from 195 patients submitted for initial biopsy because of PSA serum levels within the gray zone. They showed that the EXO106 score (the sum of normalized exosomal *PCA3* and *ERG* mRNA levels) is related to the Gleason score, showing higher results in patients with a Gleason score ≥7. The authors proposed to combine the EXO106 score with PSA, age, race and PCa family history for the detection of high PCa, showing negative and positive predictive values of 97.5% and 34.5%, respectively (AUC: 0.803).

Several authors suggested that exosomes obtained from blood and urine are a consistent source of miRNA for disease biomarker detection [85,86,87,88], although some doubts have been presented by other researchers underlying that exosomes in standard preparations do not carry a biologically significant amount of miRNAs [89]. Moreover, according to Arroyo et al. [90], vesicle associated miRNAs only represent a minority, while around 90% of miRNAs in the circulation are present in a non-membrane-bound form. Instead, Gallo et al. [91] showed that the majority of miRNAs detectable in serum and saliva are concentrated in exosomes. Moreover, Cheng et al. [85] showed that in urine the highest proportion of miRNA was extracted from exosomes.

Actually, some studies have evaluated the usefulness of exosomes’ miRNAs in PCa management (Table 1). Li et al. [92] showed that the level of the miR-141 was significantly higher in exosomes compared with the whole serum. Also, these authors reported that the level of serum exosomal miR-141 was significantly higher in PCa patients compared with BPH patients and healthy controls, finding the most elevated levels in patients with metastatic PCa. Moreover, Huang et al. [93] found that the levels of plasma exosomal miR-1290 and miR-375 were significantly associated with poor overall survival. The addition of these new biomarkers into a clinical prognostic model improved predictive performance with a time-dependent AUC increase from 0.66 to 0.73. On the other hand, Bryzgunova et al. [94] demonstrated promising results in the detection of PCa, analyzing miR-19b in urinary exosomes isolated by differential centrifugation. Finally, Samsonov et al. [95] indicated that miR-21, miR-141 and miR-574 were upregulated in PCa patients compared with healthy controls in urinary exosomes isolated by a lectin-based exosomes agglutination method. Nevertheless, only miR-141 was found to be significantly upregulated when urinary exosomes were isolated by differential centrifugation.

More recently, NGS technologies revealed the presence of sequence variants of miRNAs, called isomiRs, showing their utility as biomarkers. They are generated from a single miRNA locus through the miRNA processing and maturation process. Koppers-Lalic et al. [96] identified that isomiRs of miR-21, miR-375 and miR-204 measured in urinary extracellular vesicles could distinguish control men from PCa patients. The authors found an AUC of 0.821 for these set of isomiRs, meanwhile the AUC corresponding to the mature miRNA was 0.661. The existence of isomiRs could explain disagreements about the usefulness of miRNAs in PCa detection.

Preliminary results about exosomal biomarkers are promising, although contradictory results have been published. New advances in standardization of isolation and characterization procedures together with larger clinical studies are required to assess the clinical usefulness of exosomes.

## 6. Conclusions

PCa is a very heterogeneous tumor, including patients with a low-risk of progression, in which cancer-specific survival rates exceeded 99% at a 15-year follow-up. The percentage of low-risk PCa has been estimated by Klotz [97] to be between 50% and 60% of new diagnosed cases. However, the survival rate decreases considerably for men with aggressive PCa. Specific gene-expression patterns have been identified for PCa subclasses. Recently, Rubin et al. [98] have reported specific genomic profiles related to the Gleason score, showing that few driver mutations and no polyploidy are associated with tumors with a Gleason score lower than 7. In this regard, to label those patients with a Gleason 6 or lower as having cancer has been put into question [99,100].

New biomarkers have been recently introduced for the management of early PCa, offering improvement in detection and some are useful in differentiating between aggressive and non-aggressive PCa. In this article, we have underlined recent advances in the discovery of PCa biomarkers related to aggressiveness. The 4kscore is defined to identify with high accuracy an individual patient’s risk for aggressive PCa. Also, high values of PHI are associated with tumor aggressiveness. Besides, both tests outperform the specificity of tPSA and %fPSA. Furthermore, although the most appropriate cut-off for the PCA3 score still has to be established, it outperforms better than tPSA and %fPSA, although its relationship with the aggressiveness of the tumor is controversial. More results are necessary to identify more accurately the usefulness of emerging biomarkers based on molecular techniques, including the *TMPRSS2:ERG* fusion gene and exosomal and non-exosomal miRNAs. Furthermore, new efforts in the standardization of these methods are necessary to use these novel biomarkers routinely.

Available studies comparing PCa biomarkers showed non-conclusive results, although several studies have evaluated these biomarkers. Scattoni et al. [101] evaluated the PHI and PCA3 score in 211 patients undergoing initial (116) or repeat (95) prostate biopsy, finding that the PHI was significantly more accurate than the PCA3 score for predicting PCa (AUC 0.70 vs. 0.59). However, Stephan et al. [102] evaluated 246 patients, showing no significant differences in accuracy between the PCA3 score and the PHI (AUC 0.74 vs. 0.68), whereas the urinary *TMPRSS2:ERG* fusion gene failed to significantly improve the ability to detect PCa. On the other hand, only PSA and the PHI correlated with the Gleason score, whereas *PCA3*, %fPSA, and the *TMPRSS2:ERG* fusion gene did not. Instead, Tallon et al. [103] concluded that the PHI and urinary *PCA3* and *TMPRSS2:ERG* fusion gene are complementary predictors of cancer aggressiveness at radical prostatectomy. According to this group, the PCA3 score was related to tumor volume ≥0.5 mL and multifocality, while the PHI was related to tumor volume ≥0.5 mL, the Gleason score ≥7 and extracapsular extension. The *TMPRSS2:ERG* fusion gene was only related to the pathological T stage.

A comparison between the 4Kscore and the PHI was provided by Nordström et al. [104] evaluating the performance of these biomarkers in a series of 531 men with PSA levels between 3 and 15 μg/L. No significant differences were found between both tests in the detection of any-grade PCa as well as in the detection of high-grade PCa (AUCs: 0.69 and 0.718, respectively, for the 4Kscore; and 0.704 and 0.711, respectively, for the PHI). On the other hand, Vedder et al. [105] compared PCA3 and the 4Kscore in 708 patients in which biopsy was done when PSA was ≥3 μg/L or PCA3 score was ≥10. The 4Kscore outperforms the PCA3 score (AUC 0.78 vs. 0.62) in men with elevated PSA, although the accuracy of the PCA3 score was higher in the global population (AUC 0.63 for PCA3 vs. 0.56 for the 4Kscore). Additionally, the authors showed that both tests increased the value of a base multivariate model (from 0.70 to 0.73 adding the PCA3 score and 0.71 adding the 4kscore), but significant differences were only found adding the PCA3 score (*p* = 0.02).

To summarize, in recent years, several new promising PCa biomarkers have been identified and found to be associated with tumor aggressiveness (Box 1). Multicenter prospective studies showed the utility of the PHI, the 4Kscore and the PCA3 score to reduce the number of unnecessary prostate biopsies in PSA tested men. Actually, these biomarkers have been recommended for different guidelines [11,106,107]. However, large prospective studies, avoiding bias due to preselection of patients according to PSA serum levels, are necessary to compare the value of these biomarkers. Also, new efforts are necessary to standardize the methodology for the measurement of exosomal and non-exosomal miRNAs to analyze accurately their usefulness in the management of patients with early PCa. Finally, the combined role of these biomarkers together with magnetic resonance imaging data would be elucidated [108,109,110]. Additionally, results obtained using blood and urinary biomarkers must be compared with promising data obtained with Prostate-specific membrane antigen (PSMA). PSMA is a transmembrane glycoprotein overexpressed in PCa cells. Results of PSMA serum levels in the detection of the PCa are inconclusive, but its expression is correlated with a higher Gleason score, PSA at diagnosis and advanced clinical stage. PSMA features enable this biomarker as an optimal target for developing imaging strategies for PCa. Recent results show that the use of PET probes targeting PSMA for imaging prostate cancer might improve detection of cancerous foci within the prostate, especially in patients with a previous negative prostate biopsy [111]. Further studies are necessary to better characterize new PCa biomarkers, but available publications show promising results to detect PCa and to distinguish patients with aggressive and non-aggressive PCa.

Box 1. **Biomarkers in PCa detection and prognosis.**

**Prostate health index**
      Biomarkers measured: PSA, fPSA, [−2]proPSA
      Sample: serum
      Approved by the Food and Drug Administration (FDA)
      Recommended by the National Comprehensive Cancer Network
      Related to PCa aggressiveness
	   
      **4Kscore**
      Biomarkers measured: PSA, fPSA, iPSA, hK2
      Sample: serum
      The test provides information about the probability of having a high-risk PCa
      Recommended by the National Comprehensive Cancer Network
      Related to PCa aggressiveness
	   
      **PCA3 score**
      Biomarkers measured: mRNA *PCA3* in relation to mRNA *PSA*
      Sample: urine obtained after prostate massage
      Approved by the Food and Drug Administration (FDA)
      Recommended by the National Comprehensive Cancer Network
      Inconclusive results about its relationship with PCa aggressiveness
	   
      ***TMPRSS2:ERG* fusion gene**
      Biomarkers measured: mRNA *TMPRSS2:ERG* in relation to mRNA *PSA*
      Sample: urine obtained after prostate massage
      Preliminary results
	   
      **miRNAs and other exosomal biomarkers**
      Sample: blood and urine
      Directly related to development and progression of cancer
      No standardized methodology
      Preliminary results

## Figures and Tables

**Table 1:** microRNAs studies in PCa patients.

Reference	Body Fluid	miRNAs Analyzed	Methodology	Patients	Clinical Results
Mitchell et al. 2008 [61]	Serum	miR-100, -125b, -141, -143, -205, and -296	qRT-PCR	25 metastatic PCa and 25 matched healthy controls	AUC of 0.907 for miR-141 comparing PCa and healthy
Mihelich et al. 2015 [63]	Serum	21 miRNAs	qRT-PCR	100 no treated PCa (50 low-grade, 50 high-grade) and 50 BPH	A panel combining let-7a, miR-103, -451, -24, -26b, -30c, -93, -106a, -223, -874, -146a, -125b, -100, -107 and -130b distinguish high-grade PCa from low-grade PCa and BPH
Chen et al. 2012 [64]	Plasma	1146 miRNAs, 8 selected miRNAs for validation study	Illumina’s Human miRNA microarray, qRT-PCR	Screening set: 17 BPH and 25 CaP. Validation set: 44 BPH, 54 healthy controls and 80 CaP	A panel combining miR-622, -1285, -30c, let-7e and let-7c discriminate CaP from BPH (AUC: 0.924) or healthy controls (AUC: 0.860)
Moltzahn et al. 2011 [65]	Serum	384 miRNAs, 12 miRs selected for validation study	multiplex qRT-PCR	12 low-risk PCa, 12 intermediate-risk PCa, 12 high-risk PCa and 12 healthy controls	AUCs: miR-106a, 0.928; miR-1274, 0.928; miR-93, 0.907; miR-223, 0.876; miR-874, 0.845; miR-1207, 0.812; miR-24: 0.778. miR-93, -106a and -24 differentiate healthy and metastatic groups
Salido-Guadarrama et al. 2016 [66]	urine obtained after prostate massage	364 miRNAs	MicroRNA TaqMan Low Density Array, qRT-PCR	73 patients with high-risk PCa and 70 patients with BPH	AUC for miR-100/200b signature was 0.738. Adding the miR-100/200b signature to a multivariate model based on age, DRE, total PSA and %fPSA the AUC increased from 0.816 to 0.876
Li et al. 2015 [92]	serum exosomes	miR-141	qRT-PCR	Serum vs. exosomes cohort: 20 PCa, 20 BPH, 20 healthy controls	Serum exosomal miR-141 was significantly higher in PCa patients compared with BPH patients and healthy controls
Huang et al. 2015 [93]	Plasma exosomes	let-7c, miR-30a/e, -99a, -1246, -1290, -16, -125a, and -375	Illumina HiSeq2000 platform, qRT-PCR	Screening cohort: 23 CRPC patients. Follow-up cohort: 100 CRPC patients	Plasma exosomal miR-1290 and miR-375 were significantly associated with poor overall survival
Bryzgunova et al. 2016 [94]	total extracellular vesicles and exosome-enriched fractions	miR-19b, -25, -125b, and -205	qRT-PCR	20 healthy controls and 14 untreated PCa patients	Detection of miR-19b versus miR-16 in total vesicles and exosome-enriched fractions achieved 100%/93% and 95%/79% specificity/sensitivity in distinguishing cancer patients from healthy individuals, respectively, demonstrating the diagnostic value of urine extracellular vesicles. miR-19b in total extracellular vesicles distinguishes cancer patients from healthy individuals with a sensitivity of 93% and a specificity of 100%
Samsonov et al. 2016 [95]	Urinary exosomes	miR-21, -107, -141, -221, -298, -326, -375, -432, -574, -2110, -625, -301a, -191	qRT-PCR	35 PCa patients and 35 healthy controls	miR-21, -141 and -574 were upregulated in PCa patients compared with healthy controls in urinary exosomes

AUC: area under the curve, BPH: benign prostatic hyperplasia, CRPC: castration resistant prostate cancer, PCa: prostate cancer.
